# *Clostridioides difficile* Infection in Special Populations: Focus on Inflammatory Bowel Disease—A Narrative Review from Pathogenesis to Management

**DOI:** 10.3390/biomedicines13112702

**Published:** 2025-11-03

**Authors:** Cristina Seguiti, Enrico Tettoni, Edoardo Pezzuto, Viviana Gerardi, Alessandro Quadarella, Paola Cesaro, Paolo Colombini

**Affiliations:** 1 SS Malattie Infettive, Fondazione Poliambulanza Istituto Ospedaliero, 25124 Brescia, Italy; 2 U.O.C. Medicina Generale e Geriatria, Fondazione Poliambulanza Istituto Ospedaliero, 25124 Brescia, Italy; 3 Gastroenterology and Digestive Endoscopy Unit, Fondazione Poliambulanza Istituto Ospedaliero, 25124 Brescia, Italy; 4 Fondazione Policlinico Universitario A. Gemelli IRCCS, Università Cattolica del Sacro Cuore, 00168 Rome, Italy

**Keywords:** *Clostridioides difficile*, inflammatory bowel diseases, ulcerative colitis, Crohn’s disease

## Abstract

*Clostridioides difficile* infection (CDI) is a major complication in inflammatory bowel disease (IBD), due to coexistence of altered microbiota, chronic inflammation, and immune dysregulation. This narrative review summarizes recent evidence on the epidemiology, pathogenesis, risk factors, diagnosis, and management of CDI in IBD. Overall, IBD patients have a four- to five-fold higher risk of CDI than the general population and face more severe courses, higher rates of hospitalization, colectomy, recurrence, and mortality. Pathogenesis involves profound dysbiosis with loss of butyrate-producing *Firmicutes* and *Bacteroidetes*, bile acid imbalance that promotes spore germination, and enhanced toxin effects on an already inflamed mucosa. Major risk factors include active colonic disease, broad-spectrum antibiotic exposure, prolonged hospitalization, and corticosteroid or combined immunosuppressive therapy. Diagnosis requires careful integration of stool assays with clinical evaluation, supported by endoscopy or imaging when needed, to distinguish infection from IBD flares. Recommended first-line treatments are fidaxomicin or oral vancomycin, reserving fecal microbiota transplantation for recurrent or high-risk cases. Optimal IBD control is essential to reduce both primary and recurrent infection. CDI and IBD share a mutual pathogenic interplay in which microbial, immune, and therapeutic factors from each condition drive and magnify the other. Early recognition, guideline-based antibiotic therapy, judicious use of immunosuppression, and microbiota-based preventive strategies are crucial to improve patient outcomes and limit recurrence, thus reducing healthcare costs.

## 1. Introduction

*Clostridioides difficile* infection (CDI) has emerged as a major challenge for healthcare systems, with a substantial impact on patient morbidity, mortality, and healthcare costs. European surveillance data confirm the increasing spread of this pathogen, which remains among the most common healthcare-associated infections. Notably, community-onset cases—defined as CDI with symptom onset outside healthcare facilities, within 48 h of hospital admission, or more than 12 weeks after discharge—are also rising, now accounting for approximately 33% of all CDI cases in Europe [1,2,3,4].

*C. difficile* is a spore-forming, Gram-positive, anaerobic bacterium capable of producing up to three exotoxins—toxin A (TcdA), toxin B (TcdB), and a binary toxin known as CDT—which constitute its principal virulence factors. Transmission occurs via the fecal–oral route through spores that germinate in the presence of primary bile salts upon reaching the small intestine. An altered gut microbiota favours migration, stable colonization, and proliferation of the vegetative form in the colon, followed by exotoxin secretion that disrupts the colonic epithelium, leading to fluid secretion, inflammation, and tissue damage [5]. The clinical spectrum of CDI ranges from mild diarrhea to fulminant colitis and toxic megacolon, the latter sometimes requiring surgical colonic resection. Although colonization with toxigenic *C. difficile* can occur and remain asymptomatic, several conditions have been identified as predisposing factors for the development of symptomatic disease. These include host-related factors such as advanced age, impaired immune status, and comorbidities; increased risk of exposure to *C. difficile* spores in the environment; and factors that disrupt the normal physiology of the gut microbiota [6].

Among the aforementioned predisposing comorbidities, inflammatory bowel disease (IBD) is of particular interest because of the close interplay between this enteric pathogen and the chronic disease’s clinical course. Crohn’s disease (CD) and ulcerative colitis (UC), which account for the majority of IBD diagnoses, have shown a steady rise in incidence worldwide. International guidelines underscore the complexity of IBD management, which often necessitates immunosuppressive and biologic therapies that may increase susceptibility to opportunistic infections [7,8]. In this context, CDI emerges as a clinically significant complication, not only because of the diagnostic challenge of distinguishing it from IBD flares but also because it is associated with more severe disease courses, higher hospitalization rates, increased risk of colectomy, and greater mortality [9,10]. Notably, studies report a 90-day CDI readmission rate of about 0.1% among patients with a prior IBD-related hospitalization, with the risk of CDI-related readmission being higher in patients with complicated or uncomplicated UC than in those with uncomplicated CD [11,12]. Studies have also shown that patients with IBD have a significantly higher risk of recurrent CDI (rCDI), tend to experience their first episode at a younger age than non-IBD patients, and face a greater risk of colectomy [13].

Despite the growing body of literature addressing CDI in the general population, current evidence remains fragmented regarding its specific impact on patients with IBD. Most available studies focus on isolated aspects such as epidemiology or treatment, often neglecting the complex bidirectional interplay between dysbiosis, immune dysregulation, therapeutic exposures, and disease outcomes. This review aims to address these knowledge gaps by critically examining the pathogenesis, epidemiology, risk factors, diagnostic challenges, and evolving treatment strategies of CDI in IBD.

## 2. Methodology

We conducted a comprehensive search across PubMed, Scopus, and Medline, including only English-language articles published up to the end of August 2025. Our search strategy utilized detailed search strings incorporating the following terms: “*Clostridioides difficile*”, “inflammatory bowel disease”, “IBD”, “ulcerative colitis”, “Crohn’s disease”, “diagnosis”, “treatment”, “antibiotics”, “vancomycin”, “fidaxomicin”, “bezlotoxumab”, “fecal microbiota transplantation”. Furthermore, we performed a manual search of the reference lists of the selected studies and related reviews to detect any other relevant publications.

## 3. Epidemiology of CDI in IBD Patients

Comparative studies consistently show that individuals with IBD have a four- to five-fold higher risk of developing CDI, which is among the most relevant infections affecting this population, leading to more frequent and prolonged hospitalizations and a higher risk of complications [14,15,16,17].

A Canadian cohort study conducted between 2005 and 2014 reported an incidence of CDI about 4.8 times higher in patients with IBD than in non-IBD subjects, with no major differences between CD and UC. Notably, IBD patients with CDI were younger, with a mean age more than 10 years lower than that of non-IBD cases (42 vs. 52 years). Acquisition settings also differed: more than 40% of CDI episodes in IBD patients were community-acquired, whereas nosocomial cases predominated among non-IBD patients, suggesting that disease-related vulnerability and immunosuppressive therapies contribute more than healthcare exposure alone [18]. Paediatric studies confirm that IBD confers vulnerability to CDI irrespective of age [19].

Temporal trends vary geographically. In the United States, analysis of the National Inpatient Sample documented a CDI prevalence of 37.3 per 1000 discharges in UC patients and 10.9 per 1000 in CD patients, compared with 4.5 per 1000 in the general population, with a steady rise from 1998 to 2004 [16]. Between 2003 and 2011, incidence and adverse outcomes increased significantly before stabilizing in subsequent years [14]. A retrospective study of IBD-related hospitalizations from 2010 to 2020 found that CDI rates increased from 1.64% to 3.32% in CD and from 4.15% to 5.81% in UC between 2010 and 2015, followed by a gradual decline to 2.27% and 4.27%, respectively, by 2020 [20].

In Europe, data are more heterogeneous. In the United Kingdom, the proportion of hospitalized IBD patients with CDI fell dramatically from 8.7% to 0.4% between 2007/08 and 2012/13, likely reflecting the impact of antimicrobial stewardship and infection-control measures [21]. In France, CDI has been reported in about 5% of IBD flares [22].

The Asian scenario differs again. In China, the prevalence of CDI among patients hospitalized with active IBD rose from less than 2% to more than 12% within five years [23]. In Korea, both adult and paediatric cohorts have shown a progressive increase, with cytomegalovirus coinfection emerging as an important driver of adverse outcomes [24].

These regional data are corroborated by a recent global meta-analysis of 28 studies encompassing nearly 800,000 IBD patients from 11 countries across three continents, which found that roughly one in ten IBD patients developed CDI, yielding an overall pooled prevalence of 8.8%. Prevalence varied by geography, reaching 11.0% in Asia and around 8% in both North America (7.85%) and Europe (7.92%) [25].

Regardless of geography or time period, CDI in IBD is consistently associated with worse outcomes, including increased in-hospital mortality: Nguyen et al. reported odds ratios of 3.9 for UC and 1.66 for CD, while Saffouri et al. documented a four-fold increase in mortality risk (OR 4.5), together with prolonged hospital stays and a higher likelihood of discharge to long-term care facilities [16,17].

## 4. Pathogenesis

**Figure 1:**
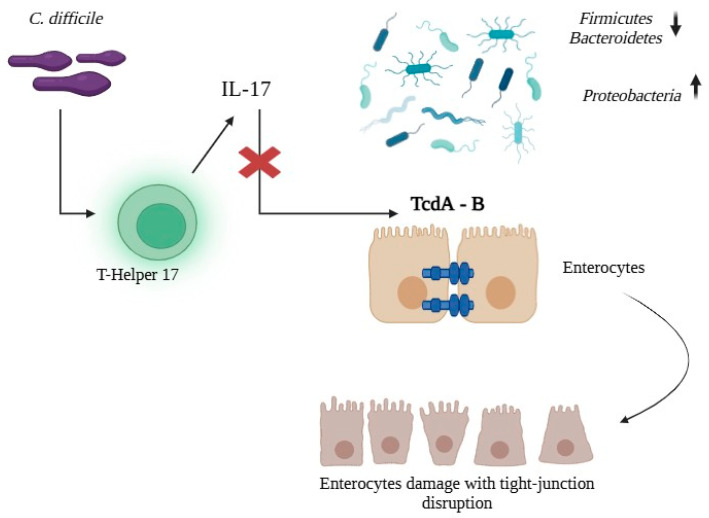
The pathogenetic interplay between CDI and IBD. Dysbiosis erodes colonization resistance, while barrier dysfunction enables TcdA/TcdB-mediated tight-junction injury. Concomitant immune skewing with heightened Th17/IL-17 activity amplifies inflammation, collectively fostering colonization, epithelial damage, and recurrence.

Multiple elements allow *C. difficile* to colonize, injure and recur, particularly in IBD setting (see Figure 1).

The characteristic dysbiosis of IBD creates a fertile environment for *C. difficile* colonization and expansion. The gut microbiota in IBD shows a marked depletion of Firmicutes—particularly *Clostridioides* clusters IV and XIVa, key butyrate producers—and of *Bacteroidetes*, alongside an overgrowth of *Proteobacteria*. This loss of protective functions compromises colonization resistance and disrupts bile acid metabolism [26]. The resulting reduction in microbial diversity not only facilitates pathogen establishment, but also enhances its pathogenic potential by diminishing metabolic competition [27]. Patients with rCDI often exhibit a bile acid profile skewed toward primary bile acids, which stimulates *C. difficile* spore germination, while secondary bile acids—normally inhibitory—are markedly reduced [28]. Epithelial barrier dysfunction provides the anatomical substrate on which toxins and a dysregulated immune response act. IBD-associated inflammation disrupts tight junctions and increases paracellular permeability, exposing the mucosa to bacterial antigens and toxins. In this context, *C. difficile* acts as a “second hit,” precipitating the breakdown of mucosal homeostasis [29]. Using a dextran sulfate sodium–induced murine colitis model, Dong et al. further clarified these mechanisms: CDI exacerbated dysbiosis by depleting protective taxa and induced pronounced neutrophil recruitment to the mucosa [30].

The host immune response is another key determinant. Even in the absence of overt infection, IBD patients exhibit abnormal immune reactivity to *C. difficile*, characterized not only by alterations in innate defense mechanisms but also by dysfunctional adaptive immunity. In particular, they show reduced numbers of pathogen-specific regulatory T cells and an overactivation of T helper 17 (Th17) responses, with excessive interleukin (IL)-17 production, which collectively impair pathogen control and predispose to exaggerated inflammatory damage. Recent immunological findings have reinforced this concept: Cook et al. (2024) [31] demonstrated that IBD patients without any documented history of *C. difficile* infection display evidence of prior, subclinical exposure to TcdB, with significantly increased circulating TcdB-specific CD4^+^ T cells compared with healthy controls. However, these responses were qualitatively dysregulated, showing a skewed T-cell phenotype with a reduced proportion of TcdB-specific Th17 cells and impaired gut-homing capacity (integrin β7 expression), suggesting ineffective mucosal immune protection against *C. difficile*. These alterations may reflect chronic occult antigenic stimulation driven by intestinal dysbiosis and support the hypothesis that immune recognition of *C. difficile* toxins contributes to ongoing inflammation in IBD, even in the absence of clinically recognized infection [31]. The ability to generate neutralizing antibodies against TcdA and TcdB strongly influences clinical outcomes, as patients unable to mount an adequate antibody response are more prone to severe and recurrent disease [32]. In rCDI defects in innate immunity, including diminished antimicrobial peptide production and impaired macrophage and dendritic cell function, further hinder spore clearance and favour chronic infection [33].

Toxin activity remains central to pathogenesis as well. *C. difficile* produces TcdA and TcdB, which disrupt the actin cytoskeleton of enterocytes, inducing apoptosis. In IBD, their effects are superimposed on an already compromised mucosa. TcdB shows enhanced cytotoxicity in the presence of proinflammatory cytokines typical of the IBD milieu (Tumor Necrosis Factor α (TNFα), Interferon-γ, IL-1β), leading to synergistic epithelial injury and further barrier breakdown [34]. Moreover, both TcdA and TcdB induce vascular endothelial growth factor A expression and increase vascular permeability, thereby promoting exudation, edema, and inflammatory cell infiltration [35]. While detrimental under normal conditions, this mechanism is particularly damaging in IBD, where mucosal vascularization is already heightened and permeable due to chronic inflammation.

## 5. Risk Factors for CDI in IBD Patients

In IBD, the risk of CDI reflects a complex interplay of disease-related factors, therapeutic exposures, and healthcare contact (see Table 1).

Colonic localization and active inflammation are the most consistent determinants of CDI risk. Severe colonic inflammation—particularly during flares and hospitalizations—promotes dysbiosis, loss of colonization resistance, and increased healthcare exposure. A French retrospective study reported that patients with colonic involvement were more likely to develop CDI (OR 2.2) [22], a finding confirmed in the meta-analysis by Balram et al. (OR 2.76) [36]. Recent prospective studies further indicate that active disease independently predicts infection, even in the absence of antibiotic use [37,38]. Across multiple cohorts, CDI has been consistently more frequent in UC than in CD, with a two- to threefold higher risk [22,38,39].

Exposure to broad-spectrum antibiotics, particularly fluoroquinolones and cephalosporins, remains one of the strongest risk factors. Jakubowska et al. reported a more than four-fold increased risk of CDI in case of antibiotic use, irrespective of IBD activity [40]. Similarly, Balram et al. found increased risk in IBD patients who had received antibiotics within 30 days of CDI testing (OR 1.85) [36]. However, a more recent meta-analysis did not confirm a statistically significant association, despite higher CDI rates in patients with prior antibiotic use [25].

Healthcare exposure also plays a central role. Zhang et al. observed that mean length of hospital stay was 10 days longer in patients with CDI, and risk increased proportionally with the duration of stay [41]. Extending hospitalization by a single day increases CDI risk by approximately 14% [40].

Immunosuppressive and immunomodulator regimens significantly influence CDI susceptibility. In a large retrospective cohort of 10,662 IBD patients treated for IBD, corticosteroid therapy tripled CDI risk compared with other immunosuppressants [42]. Other series confirmed more than a three-fold risk increase for corticosteroids [18,43]. Evidence for traditional azathioprine, 6-mercaptopurine and methotrexate is mixed: some studies show no independent risk, whereas others find significance only when these drugs were combined with steroids or biologics.

As for newer agents, anti-TNFα biologics were not associated with CDI in some studies [32,44]. However, Balram’s meta-analysis found an increased risk of CDI in patients exposed to these drugs (OR 1.65) [36]. Pivotal vedolizumab trials did not signal excess risk [45], yet a meta-analysis of 30 comparative studies reported a pooled risk ratio of 0.15 in UC and 1.29 in CD compared with anti-TNFα agents [46]. Ustekinumab has not shown a significant CDI risk in pooled analyses [47], and tofacitinib likewise did not demonstrate an increased incidence [48]. Overall, the impact of advanced therapies for IBD appears smaller than that of steroids and antibiotics, but combination therapy has been associated with a three-fold higher risk [39].

rCDI is also more frequent in IBD than in the general population. The RECIDIVISM study reported a 32% recurrence rate in IBD patients versus 24% in non-IBD controls [13]. Recent antibiotic use, 5-aminosalicylic acid therapy, steroids, and biologics all increased rCDI risk. Cohort and registry studies further identify repeated hospitalizations, cumulative immunosuppressive burden (combination therapy or rapid treatment escalation), and severe baseline endoscopic activity as key predictors of recurrence [18,24,37,39,49]. These findings highlight the importance of optimal disease control not only to prevent primary CDI, but also to reduce relapses.

**Table 1:** Summary of evidence on risk factors for CDI in IBD.

Author, Year	Study Design	Population	IBD Type	Risk Factors for CDI (Main Results)
Schneeweiss et al., 2009 [42]	Population-based cohort	Adults with IBD (n = 10,662)	UC and CD	Corticosteroids (three-fold risk, RR 3.38; dose/duration independent)
Regnault et al., 2014 [22]	Retrospective cohort	Hospitalized IBD flares (n = 813)	UC and CD	Recent intake NSAIDs (OR 3.8) independent predictor of CDI (within 2 months prior admission)
Zhang et al., 2016 [41]	Retrospective cohort	Hospitalized IBD (n = 646)	UC and CD	CD: fistula (OR 2.48), antibiotic use (OR 5.11), infliximab use (OR 2.22); synergy when antibiotics + infliximab (risk increased 10.2-fold, *p* < 0.001. UC: infliximab use (OR 2.60)
Razik et al., 2016 [13]	Retrospective cohort	Hospitalized rCDI (n = 503, IBD = 110)	UC and CD	rCDI: 5-ASA (HR 2.15), non-ileal CD (HR 2.85). IBD 33% higher rates rCDI versus non-IBD
Balram et al., 2019 [36]	Systematic review & meta-analysis	Pooled IBD studies (n = 38,336 IBD with CDI, n = 1,199,752 IBD without CDI; n = 22 observational studies)	UC and CD	Colonic involvement in CD (OR 2.76); antibiotics ≤ 30 days (OR 1.85); biologics (OR 1.65). Higher colectomy risk (OR 2.22)
Chen et al., 2019 [38]	Prospective cohort	Hospitalized IBD (n = 230)	UC and CD	UC: Longer disease, hospitalization within previous 3 months, proton pump inhibitor use within 1 month, severe disease activity (*p* < 0.05) Crohn’s disease: Moderate disease activity (*p* = 0.03) Increased surgery and colectomy rate
Voth et al., 2021 [49]	Retrospective cohort	IBD with CDI (n = 137)	UC and CD	Overweight BMI (OR 2.85); statin use (OR 5.66) linked to severe/complicated CDI
Sandborn et al., 2021 [47]	Pooled phase 2/3 trials Ustekinumab	IBD receiving Ustekinumab (n = 2574)	UC and CD	No significant increase in CDI vs placebo over 1 year
Song et al., 2023 [24]	Nationwide population-based cohort	N = 54,836 IBD vs n = 109,178 controls	UC and CD	Age (aHR 1.02/yr), female (aHR 1.46), Charlson Comorbidity Index ≥ 3 (aHR 1.50), 5-ASA (aHR 1.64), immunomodulators (aHR 1.83), biologics (aHR 2.51), long-term steroids > 90 days (aHR 1.40) Risk of CDI 7 times higher in IBD vs non-IBD
Loftus et al., 2023 [48]	Pooled RCTs + long-term extension Tofacitinib	IBD receiving tofacitinib (n = 1157)	UC	Low overall CDI incidence rate (0.31; no significant excess vs placebo)
Jakubowska et al., 2024 [40]	Single-center retrospective	Hospitalized IBD (n = 204)	UC and CD	Low BMI (*p* < 0.001); Broad-spectrum antibiotics (OR 4.86), steroids (OR 3.62), azathioprine (OR 3.39)
Chen et al., 2024 [46]	Systematic review & meta-analysis Vedolizumab	IBD receiving vedolizumab (n = 41,862; 30 studies)	UC and CD	No additional risk of CDI with vedolizumab CDI rates are higher in UC than CD (RR 2.25)
Martínez-Lozano et al., 2025 [39]	Retrospective cohort	IBD ± rheumatologic diseases (n = 1866, IBD n = 1041)	UC and CD	IBD (OR 18.29), UC (OR 2.00), ≥3 different biologic agents received (OR 3.09)
Vitikainen et al., 2025 [37]	Nationwide registry	IBD with CDI (n = 279)	UC and CD	Higher IBD activity (*p* < 0.001), short disease duration (<2 years; *p* < 0.001), UC and colonic CD (*p* = 0.001), systemic corticosteroid use (within 3 months, *p* < 0.001), hospitalization (previous 3 months, *p* < 0.001), antibiotic use (*p* < 0.001) and proton pump inhibitors (*p* < 0.001)
Amakye et al., 2025 [25]	Systematic review & meta-analysis	796,244 IBD (28 studies)	UC and CD	Male sex (OR 1.18), older age (OR 1.06); highest prevalence observed in Asia (11%)

## 6. Diagnosis

### 6.1. Clinical Assessment

While in non-IBD patients American guidelines recommend to search for CDI in patients with at least 3 bowel movements per day with unformed stools [10], the clinical features of CDI in IBD population is more complex. Given that the rates of bowel surgery in IBD patients, though decreasing, are still relatively high [50], an important distinction on CDI presentation should be based upon the bowel anatomy.

Subjects with intact intestinal anatomy present with a variety of symptoms, including diarrhea, hematochezia, worsening abdominal pain, or fever [51]. Because of the overlap in clinical presentation between CDI and IBD flare, which in turn may occur concomitantly with *C. difficile* colonization, IBD patients must be routinely checked for CDI when presenting with the aforementioned symptoms.

On the other hand, IBD patients with medically refractory colonic IBD or with colonic dysplasia/neoplasia undergo surgery. In case of UC, the first surgical option is restorative proctocolectomy, a 3-step surgery that leads to the formation of an ileal pouch–anal anastomosis (IPAA) [52]. In patients with CD, surgery consists of total proctocolectomy with permanent end ileostomy [53]. Patients without a colon can have a symptomatic CDI as well. While the pathogenesis of CDI in other bowel segments is less understood, the underlying mechanisms seem to resemble those of colonic infection [54]. Interestingly, a large database study of patients hospitalized with pouchitis (inflammation of the IPAA) found that CDI appears to be limited to those patients in which the indication to surgery was IBD, instead of other conditions (e.g., familial adenomatous polyposis) [55].

CDI in patients with pouch may be indistinguishable from the clinical onset of pouchitis, which is defined as an increase in the frequency of stools or abdominal symptoms. Most patients experience resolution of these symptoms after empiric treatment with antibiotics (usually ciprofloxacin or metronidazole). When these treatments fail to contain the symptoms, pouchitis is defined as chronic antibiotic-resistant. Patients affected by this condition may additionally develop fevers, leukocytosis, or weight loss. Treatment options range from prolonged antibiotic therapy to corticosteroids to biologic therapy [56]. Case series have described that most patients with CDI of the pouch were previously treated for chronic antibiotic-resistant pouchitis, and improved only after receiving a proper antibiotic treatment for CDI [57].

As for patients with an end ileostomy, the clinical manifestation of CDI is nonspecific as well, overlapping with other small bowel infections (viral or bacterial, irrespectively) and with a flare of small bowel CD. Consequently, high-volume ostomy output, abdominal pain or fever mandate for CDI testing [58].

### 6.2. Blood and Stool Examinations

Laboratory testing for CDI should be limited to symptomatic patients with clinically relevant diarrhea, usually defined as three or more unformed stools within 24 h, since examining formed samples increases the likelihood of false positives [10].

Historically, toxigenic culture and cell culture cytotoxicity neutralization assays were considered gold standards because of their excellent sensitivity in detecting toxigenic strains and their cytopathic effects. However, these techniques are now largely confined to reference laboratories due to high cost, technical complexity and long turnaround times, often extending over several days [59,60].

Therefore, more rapid and practical methods have gained prominence in routine diagnostics. Among these, enzyme immunoassays (EIAs) for TcdA and TcdB are widely adopted because of their accessibility and rapidity. However, their sensitivity is hampered by toxin instability in stool samples, resulting in potential false negatives when used as stand-alone tests. Conversely, nucleic acid amplification tests (NAATs), including PCR, provide highly sensitive and rapid detection of toxin genes, but they cannot distinguish between active infection and asymptomatic colonization. Similarly, glutamate dehydrogenase (GDH) assays serve as efficient, sensitive, and inexpensive screening tools, but as GDH is expressed by both toxigenic and non-toxigenic strains, confirmatory testing is mandatory [61].

To overcome the limitations of individual assays, both European and American guidelines advocate multistep diagnostic algorithms that balance sensitivity and specificity. The preferred approach is initial screening with a highly sensitive test—either GDH or NAAT—followed by a highly specific EIA for TcdA/TcdB if the screening test is positive. A positive screening test combined with a positive toxin assay supports the diagnosis of CDI and justifies treatment. Discordant results usually indicate colonization rather than infection, prompting clinicians to investigate alternative causes of diarrhea. In such context, results must always be interpreted in the context of clinical symptoms; confirmatory NAAT or careful clinical judgment before initiating therapy are recommended [10]. This scenario is especially problematic in IBD patients who have higher rates of asymptomatic carriage. Clinical outcome data show that patients who are NAAT-positive but toxin EIA-negative (NAAT+/Toxin−) have lower rates of CDI recurrence and similar or better outcomes compared to NAAT+/Toxin+ patients, and often do not benefit from CDI-specific therapy unless clinical suspicion is high [62,63]. In IBD populations, recent cohort studies confirm that antibiotic treatment of NAAT+/Toxin− cases does not improve IBD outcomes and may increase the risk of medication escalation and hospitalization, supporting the need for careful clinical correlation and avoidance of unnecessary treatment [64,65]. Consistent with these concerns, Cook et al. demonstrated that IBD patients without recognized CDI can exhibit heightened circulating TcdB-specific CD4^+^ T-cell responses despite negative stool tcdB PCR, suggesting occult toxin exposure and supporting targeted testing when disease activity changes [31].

Beyond pathogen-directed assays, additional laboratory markers contribute to assessing disease severity and prognosis. Leukocytosis exceeding 15,000/µL and elevated serum creatinine above 1.5 mg/dL are established markers of severe disease, while hypoalbuminemia is associated with higher risk of both complications and recurrence [66,67].

### 6.3. Endoscopy and Imaging

Endoscopy and imaging provide important adjunctive tools in the diagnosis and management of CDI, particularly in patients with severe disease, atypical presentations, or ongoing diagnostic uncertainty.

Endoscopic evaluation with flexible sigmoidoscopy or colonoscopy is not recommended for routine use because of the risk of perforation or bleeding, especially in fulminant colitis, but it can be decisive when stool assays are negative despite persistent symptoms or when differential diagnoses must be excluded. The characteristic finding is pseudomembranous colitis, with whitish or yellowish plaques of fibrin, necrotic cells, mucus, and inflammatory infiltrates covering the mucosa. However, pseudomembranes are seen in only 40–60% of cases and may also occur in other infectious or noninfectious forms of colitis [68,69]. The scenario is even more challenging in patients with IBD, in whom pseudomembranes are rarely detected—likely due to altered mucosal immunity or immunosuppressive therapies—and endoscopic appearance often overlaps with an IBD flare. In this context, biopsies obtained during lower endoscopy are useful not so much for confirming CDI, but for ruling out alternative causes of diarrhea, such as cytomegalovirus infection, ischemic colitis, or amoebiasis, and for assessing the extent and severity of IBD activity [70].

Imaging modalities serve as tools for identifying CDI complications. Plain abdominal radiographs can detect bowel dilatation, loss of haustration or free air, providing early recognition of toxic megacolon or perforation. Computed tomography (CT) is more sensitive and reveals typical features such as colonic wall thickening, pericolonic fat stranding and ascites, while also identifying complications like abscesses or free intraperitoneal gas [71]. CT is particularly valuable in patients presenting with abdominal distension, fever, ileus or hemodynamic instability, where rapid exclusion of toxic megacolon—defined radiologically as colonic dilatation exceeding 6 cm—and bowel perforation is critical for timely intervention and surgical referral [72]. Abdominal ultrasonography, though less frequently employed in this setting, may provide supportive data regarding bowel wall thickness, dehaustration, and peritoneal fluid collections, and can help determine disease extent in a noninvasive manner.

## 7. Treatment

### 7.1. IBD Management in CDI

Management of IBD in the setting of CDI requires a stepwise strategy that separates persistent infection from inflammatory activity and times immunosuppressive/immunomodulatory therapy judiciously.

Initial management prioritizes appropriate CDI antibiotics, followed by a short, predefined reassessment window for IBD control: in case of IBD flare, the American Gastroenterology Association suggests initiating corticosteroids or other immunosuppression 72–96 h after starting CDI antibiotics when colitic symptoms persist [73]. Evidence regarding the safety of early immunosuppression is mixed: retrospective series associate steroid escalation with higher risks of colectomy and prolonged hospitalization [74]. A European cohort reported a composite of serious outcomes (death, colectomy, megacolon, perforation, shock, or respiratory failure) in 12% of patients treated with combined antibiotics plus immunosuppressants versus 0% with antibiotics alone [75]. Conversely, other cohorts found no excess adverse outcomes with corticosteroids [66,75,76], and a multicenter study of 207 patients observed reduced adjusted odds of death, sepsis and colectomy within 90 days among those escalated to corticosteroids or biologics after antibiotic initiation [77]. Consistent with this uncertainty, European and British guidelines state that the effect of immunosuppressive agents on CDI progression in IBD remains unclear and that continuation or escalation should follow individualized risk assessment [78,79]. Surveys of gastroenterologists reflect this divergence, with practice split roughly evenly between antibiotics alone (54%) versus combined with steroids/immunosuppression (46%) [73].

In practice, an acceptable algorithm is to (i) confirm CDI and promptly initiate effective antibiotics; (ii) perform early endoscopic assessment when the clinical course is refractory or atypical; (iii) reassess after 72–96 h and, if colitic symptoms persist without evidence of uncontrolled infection or alternative pathology, initiate or escalate immunosuppressive/immunomodulatory therapy with vigilant monitoring; (iv) individualize decisions in light of comorbidity-driven risk [69].

### 7.2. Antibiotics

Antibiotic therapy for CDI has shifted decisively over the past decade toward regimens that maximize clinical cure while minimizing recurrence and collateral damage to the gut microbiota.

American and European guidelines now prioritize fidaxomicin over vancomycin for an initial episode and up to a first recurrence when available, while accepting oral vancomycin as a valid alternative, particularly when access or cost are limited. In non-severe and severe non-fulminant disease, recommended regimens are fidaxomicin 200 mg twice daily for 10 days or vancomycin 125 mg orally four times daily for 10 days [9,10]. Metronidazole is no longer first-line because of inferior effectiveness and higher failure/recurrence rates, and is generally reserved for settings in which fidaxomicin and vancomycin are not readily available [80,81]. In fulminant colitis (hypotension, ileus or megacolon), high-dose vancomycin 500 mg orally or via nasogastric tube four times daily plus intravenous (IV) metronidazole 500 mg every 8 h is advised; early resuscitation and surgical consultation are essential given the risk of toxic megacolon and perforation. Notably, ESCMID allows consideration of fidaxomicin and addition of IV tigecycline in severe–complicated disease and discourages IV metronidazole [9], as data supporting dual high-dose vancomycin plus IV metronidazole are mixed [82,83].

Vancomycin, a bactericidal agent, is minimally absorbed when given orally, achieving very high colonic concentrations and blocking sporulation [84]. Fidaxomicin—a bactericidal agent with minimal systemic absorption and a narrow spectrum—achieves high intraluminal concentrations, perturbs the microbiome less than vancomycin [84], and consistently reduces recurrence risk at 28 days by roughly 10–15% versus vancomycin [84,85]; pooled analyses show longer sustained remission with fidaxomicin (hazard ratio 1.16) [86]. An extended-pulsed fidaxomicin (EP-FDX) schedule (200 mg twice daily on days 1–5, then every other day on days 7–25) further improves sustained cure and delivered the lowest recurrence rates observed in randomized studies [87]. Pharmacokinetic work in IBD indicates no excess systemic absorption and consistently therapeutic fecal levels with fidaxomicin [88]. Beyond efficacy, fidaxomicin lowers vacomycin-resistant *Enterococci* and *Candida* overgrowth relative to vancomycin [89]. However, its price remains a barrier despite cost-effectiveness analyses [90,91].

Because recurrence drives morbidity, regimens that reduce relapse are prioritized. Standard practice defines recurrence as return of symptoms with a positive stool test within 8 weeks of completing appropriate therapy. For a first recurrence, therapy should differ from the index regimen: after standard-dose vancomycin, a pulsed-tapered vancomycin course (e.g., 125 mg QID for 10–14 days, then BID 7 days, then daily 7 days, then every 2–3 days for 2–8 weeks) or a 10-day fidaxomicin course are appropriate; after initial fidaxomicin or metronidazole, a 10-day standard vancomycin course is reasonable. For multiple recurrences, options include prolonged vancomycin tapers, “rifaximin chaser” (400 mg TID for 20 days after a 10-day vancomycin course), a 10-day fidaxomicin course, EP-FDX continuation after initial fidaxomicin, and escalation to fecal microbiota transplantation (FMT) [9,10].

*C. difficile* is commonly resistant to many broad-spectrum antibiotics in routine use—especially clindamycin, macrolides, fluoroquinolones, and β-lactams [92,93,94]. As for first-line CDI agents, while resistance remains uncommon at the population level, surveillance signals of reduced susceptibility are increasing [95,96,97]. For metronidazole, pooled resistance prevalence is about 1.9–5%, with rises reported among epidemic ribotypes and in high antibiotic-pressure settings [92,98]. Mechanistically, resistance stems from *nimB* mutations (encoding a nitroreductase that inactivates the drug) and acquisition of the pCD-METRO plasmid, which can raise minimum inhibitory concentrations (MICs) up to 25-fold and has been detected worldwide in both toxigenic and non-toxigenic strains [99,100,101]. Additional chromosomal changes affecting iron homeostasis/oxidoreductase pathways reduce intracellular iron and shift metabolism, thereby diminishing bactericidal activity [102]. For vancomycin, U.S. surveillance indicates elevated MICs are rare [103]. Resistance relates to activation of the usually silent vanGCd cluster via *vanSR* mutations, yielding low-level resistance; plasmid acquisition can also decrease susceptibility in vitro and in animal models, pointing to horizontal gene transfer [104,105]. Biofilm formation and recurrent CDI further reduce vancomycin efficacy, with biofilm-associated MICs up to 100× values [106]. Although high-level resistance can emerge rapidly under antibiotic pressure, associated fitness costs may limit spread [107]. Fidaxomicin resistance remains rare [108], but reduced susceptibility (MIC ≥ 2 µg/mL) and occasional clinical failures have been reported, including on-therapy selection of resistant isolates [109,110]. The principal mechanism involves *rpoB* mutations in the RNA polymerase β subunit (notably V1143) that alter the fidaxomicin binding site [111,112]; such mutations can arise after a single treatment course and often impose a fitness cost [109,110]. While high intraluminal drug concentrations likely blunt clinical impact, some isolates may develop compensatory mechanisms, underscoring the need for ongoing resistance surveillance and antimicrobial stewardship [113].

### 7.3. Fecal Microbiota Transplantation (FMT)

FMT—the engraftment of processed stool from rigorously screened healthy donors to reconstitute a dysbiotic gut ecosystem—has evolved to a guideline-endorsed therapy for recurrent or refractory CDI, including in patients with IBD. European guidelines recommend FMT after at least two recurrences, or earlier in patients with a first recurrence judged at high risk of further relapse or with severe–complicated disease [9]. Randomized and controlled data now extend beyond salvage therapy: a double-blind Danish trial of “early FMT” demonstrated superiority to vancomycin alone for sustained resolution after a first or second CDI episode [114], prompting consideration of earlier positioning in selected patients. Across delivery routes—capsules, nasoenteric tubes, upper endoscopy, colonoscopy, flexible sigmoidoscopy, and enema—no single method is universally preferred. A systematic review suggests colonoscopic administration may yield the highest cure, while capsule-based FMT has been found non-inferior to colonoscopy and appears effective at both lower (10 capsules, single administration) and higher (30 capsules) doses; patients receiving upper-GI delivery should remain upright for ≥4 h to minimize aspiration risk [115,116].

Mechanistically, FMT restores bile-acid biotransformation (re-establishing conversion of primary to secondary bile acids that suppress *C. difficile* germination), boosts short-chain fatty acids—especially butyrate—to support epithelial integrity and reduce permeability, enhances bactericidal peptide production and modulates mucosal immunity (including T-cell activity, leukocyte adhesion, and inflammatory cytokine profiles). These processes converge to reduce recurrence and heal inflamed mucosa [117].

Observational and trial data in non-IBD populations consistently show high effectiveness, with per-protocol cure rates frequently approaching 90% [118,119], while real-world series underscore the value of repeated or “sequential” FMT when a single infusion fails [120]. In IBD, early concerns about safety and efficacy under immunosuppression have been tempered by multiple cohorts: CDI cure after a single FMT commonly ranges 74–84%, rising to approximately 90% with repeat administration. Pooled estimates suggest a modest risk of IBD worsening (22.7% overall after FMT for CDI), which attenuates to a marginal signal (4.6%) when restricted to higher-quality studies and randomized trials [121]. Comparative cohorts report broadly similar CDI outcomes in IBD versus non-IBD, with 30–50% of IBD patients experiencing clinical improvement and a minority experiencing flares requiring escalation. Flare rates are heterogeneous across studies (17–25%, with UC flaring more than CD) [122,123], emphasizing the need for careful peri-procedural disease activity assessment. IBD patients with active extensive colitis or primary sclerosing cholangitis may have higher flare risk despite FMT [124]. Conversely, safety signals remain uncommon: isolated reports describe bacteremia (including two cases of ESBL-producing *Escherichia coli* traced to a single donor in non-IBD recipients), and one case series of CD-related bacteremia likely reflected baseline mucosal barrier disruption rather than FMT per se [125,126]. Current donor-screening frameworks mitigate infectious risks, and unrelated donors are generally favoured to avoid shared genetic/environmental microbiome determinants. In severe–fulminant CDI, evidence is mixed: meta-analytic synthesis suggests FMT can be effective but often requires multiple procedures plus adjunctive antibiotics [127].

A new single-dose, microbiota-based live biotherapeutic to prevent rCDI (REBYOTA^®^) warrants attention. Its performance in patients with IBD has been evaluated in a prespecified subgroup of the phase 3, open-label PUNCH CD3-OLS program. Adults received one 150 mL rectal dose 24–72 h after completing standard-of-care (SoC) antibiotics, with an optional second course for early nonresponse. Week-8 treatment success was 78.9% in the IBD group—with no significant differences between UC and CD—versus 73.2% in non-IBD. Among week-8 responders, sustained clinical response through 6 months reached 91.1% in IBD, comparable to 91.0% in non-IBD. A second course was used in 12.7% and 18.5%, respectively. Safety profiles were similar: treatment-emergent adverse events occurred in 45.9% (IBD) and 47.5% (non-IBD), predominantly mild–moderate gastrointestinal, with infrequent serious events [128].

Finally, data hint that successful CDI eradication by FMT can lessen IBD activity and improve responsiveness to standard IBD therapies [129], though robust randomized, disease-activity–stratified trials with long-term follow-up are needed to refine patient selection, optimize sequencing with antibiotic regimens and standardize outcome measures and donor protocols.

### 7.4. Monoclonal Antibodies

Monoclonal antibody therapy for CDI centered on bezlotoxumab, a fully human IgG1 directed against TcdB that was approved in 2016 as an adjunct to SoC antibiotics to reduce recurrence in adults at elevated risk. Mechanistically, bezlotoxumab neutralizes TcdB and, in vitro, attenuates toxin-driven inflammatory signaling (including monocyte activation and TNFα/IL-1β expression) and epithelial injury, thereby interrupting the pathogenic cascade without perturbing the intestinal microbiota [130].

In the double-blind, randomized MODIFY I/II trials, the 12-week recurrence rate was reduced from 28% to 17% and from 26% to 16%, respectively, when bezlotoxumab (single 10 mg/kg infusion) was added to SoC, with signals of greatest benefit in high-risk cohorts (age ≥ 65 years, prior CDI, severe presentation, immunocompromise) [131]. Pharmacologically, bezlotoxumab exhibits an elimination half-life of about 18 days, yields measurable serum concentrations for months after a single dose, requires no dose adjustment in renal or hepatic impairment, and has no known drug–drug interactions. Infusion-related reactions are the most frequent adverse events (approximately 10%) and overall tolerability mirrors placebo. Particular caution was advised in patients with pre-existing heart failure given post-marketing signals of exacerbation [130,131].

Guideline positioning reflected these data: recommendations incorporated bezlotoxumab as an adjunct for patients with initial CDI who carry risk factors for recurrence and for those with second or subsequent recurrences [9,132]. Despite this body of evidence, bezlotoxumab was discontinued from its drugmaker and retired from the market in January 2025.

Actoxumab is a fully human monoclonal antibody targeting TcdA [133]. Although actoxumab demonstrated in vitro and animal model efficacy in neutralizing TcdA and preventing toxin-mediated cellular damage, clinical trial data from the MODIFY I and II studies showed that actoxumab alone did not reduce the rate of recurrent CDI compared to placebo, and its development was discontinued after interim analysis [134].

New evidence is developing on a next-generation anti–TcdB monoclonal antibody. In a piglet model that recapitulates clinical and pathological features of human *C. difficile* infection, AZD5148 (0.5 mg/kg intraperitoneally, administered 24 h before oral challenge with a hypervirulent *C. difficile* strain) significantly attenuated disease severity—lower stool/diarrhea scores, reduced macroscopic colonic injury, and diminished epithelial damage and inflammation. These preclinical findings support further evaluation of AZD5148 as a prophylactic or early-intervention strategy in CDI [135].

## 8. Conclusions

The interplay of IBD-associated dysbiosis, mucosal barrier dysfunction, and immune dysregulation creates a biologically permissive environment for *C. difficile* colonization and for symptomatic infection, while frequent exposures to antibiotics, prolonged hospitalization, and immunosuppressive therapies further amplify risk. Epidemiological data highlight higher incidence and recurrence rates in IBD compared with the general population, with significant impacts on morbidity, hospitalization and mortality. Early and accurate diagnosis is essential to reduce the impact of CDI in IBD patients and for distinguishing infection from IBD flare. Current evidence supports a stepwise therapeutic strategy centred on fidaxomicin or vancomycin, reserving FMT and bezlotoxumab in high-risk cases. Optimal timing and intensity of immunosuppression in CDI-IBD still remain uncertain, underscoring the need for further studies addressing risk stratification, early immunosuppression, head-to-head or sequencing trials of antibiotic, antibody, and microbiota-based strategies in IBD subsets, and standardized outcomes integrating infection control, IBD activity, and quality of life.

## Data Availability

No new data were created or analyzed in this study. Data sharing is not applicable to this article.

## References

[1] Alexiou S., Diakou A., Kachrimanidou M. (2025). The Role of *Clostridioides difficile* Within the One Health Framework: A Review. Microorganisms.

[2] Dubberke E.R., Olsen M.A. (2012). Burden of *Clostridium difficile* on the Healthcare System. Clin. Infect. Dis..

[3] Kwon J.H., Olsen M.A., Dubberke E.R. (2015). The Morbidity, Mortality, and Costs Associated with *Clostridium difficile* Infection. Infect. Dis. Clin. N. Am..

[4] European Centre for Disease Prevention and Control (2019). European Surveillance of Clostridioides (Clostridium) difficile Infections: Surveillance Protocol Version 2.4.

[5] Kordus S.L., Thomas A.K., Lacy D.B. (2022). *Clostridioides difficile* Toxins: Mechanisms of Action and Antitoxin Therapeutics. Nat. Rev. Microbiol..

[6] Eeuwijk J., Ferreira G., Yarzabal J.P., Robert-Du Ry Van Beest Holle M. (2024). A Systematic Literature Review on Risk Factors for and Timing of *Clostridioides difficile* Infection in the United States. Infect. Dis. Ther..

[7] Raine T., Bonovas S., Burisch J., Kucharzik T., Adamina M., Annese V., Bachmann O., Bettenworth D., Chaparro M., Czuber-Dochan W. (2022). ECCO Guidelines on Therapeutics in Ulcerative Colitis: Medical Treatment. J. Crohn’s Colitis.

[8] Gordon H., Minozzi S., Kopylov U., Verstockt B., Chaparro M., Buskens C., Warusavitarne J., Agrawal M., Allocca M., Atreya R. (2024). ECCO Guidelines on Therapeutics in Crohn’s Disease: Medical Treatment. J. Crohn’s Colitis.

[9] Van Prehn J., Reigadas E., Vogelzang E.H., Bouza E., Hristea A., Guery B., Krutova M., Norén T., Allerberger F., Coia J.E. (2021). European Society of Clinical Microbiology and Infectious Diseases: 2021 Update on the Treatment Guidance Document for *Clostridioides difficile* Infection in Adults. Clin. Microbiol. Infect..

[10] Johnson S., Lavergne V., Skinner A.M., Gonzales-Luna A.J., Garey K.W., Kelly C.P., Wilcox M.H. (2021). Clinical Practice Guideline by the Infectious Diseases Society of America (IDSA) and Society for Healthcare Epidemiology of America (SHEA): 2021 Focused Update Guidelines on Management of *Clostridioides difficile* Infection in Adults. Clin. Infect. Dis..

[11] Palacios Argueta P., Salazar M., Attar B., Simons-Linares R., Shen B. (2021). 90-Day Specific Readmission for *Clostridium difficile* Infection After Hospitalization with an Inflammatory Bowel Disease Flare: Outcomes and Risk Factors. Inflamm. Bowel Dis..

[12] Ricciardi R., Ogilvie J.W., Roberts P.L., Marcello P.W., Concannon T.W., Baxter N.N. (2009). Epidemiology of *Clostridium difficile* Colitis in Hospitalized Patients with Inflammatory Bowel Diseases. Dis. Colon Rectum.

[13] Razik R., Rumman A., Bahreini Z., McGeer A., Nguyen G.C. (2016). Recurrence of *Clostridium difficile* Infection in Patients with Inflammatory Bowel Disease: The RECIDIVISM Study. Am. J. Gastroenterol..

[14] Ananthakrishnan A.N., McGinley E.L., Saeian K., Binion D.G. (2011). Temporal Trends in Disease Outcomes Related to *Clostridium difficile* Infection in Patients with Inflammatory Bowel Disease. Inflamm. Bowel Dis..

[15] Gillespie W., Marya N., Fahed J., Leslie G., Patel K., Cave D.R. (2017). *Clostridium difficile* in Inflammatory Bowel Disease: A Retrospective Study. Gastroenterol. Res. Pract..

[16] Nguyen G.C., Kaplan G.G., Harris M.L., Brant S.R. (2008). A National Survey of the Prevalence and Impact of *Clostridium difficile* Infection Among Hospitalized Inflammatory Bowel Disease Patients. Am. J. Gastroenterol..

[17] Saffouri G., Gupta A., Loftus E.V., Baddour L.M., Pardi D.S., Khanna S. (2017). The Incidence and Outcomes from *Clostridium difficile* Infection in Hospitalized Adults with Inflammatory Bowel Disease. Scand. J. Gastroenterol..

[18] Singh H., Nugent Z., Yu B.N., Lix L.M., Targownik L.E., Bernstein C.N. (2017). Higher Incidence of *Clostridium difficile* Infection Among Individuals with Inflammatory Bowel Disease. Gastroenterology.

[19] Hourigan S.K., Oliva-Hemker M., Hutfless S. (2014). The Prevalence of *Clostridium difficile* Infection in Pediatric and Adult Patients with Inflammatory Bowel Disease. Dig. Dis. Sci..

[20] Spartz E.J., DeDecker L.C., Fansiwala K.M., Noorian S., Roney A.R., Hakimian S., Sauk J.S., Chen P., Limketkai B.N. (2024). Recent Trends and Risk Factors Associated with *Clostridioides difficile* Infections in Hospitalized Patients with Inflammatory Bowel Disease. Aliment. Pharmacol. Ther..

[21] Joshi N.M., Marks I.H., Crowson R., Ball D., Rampton D.S. (2017). Incidence and Outcome of *Clostridium difficile* Infection in Hospitalized Patients with Inflammatory Bowel Disease in the UK. J. Crohn’s Colitis.

[22] Regnault H., Bourrier A., Lalande V., Nion-Larmurier I., Sokol H., Seksik P., Barbut F., Cosnes J., Beaugerie L. (2014). Prevalence and Risk Factors of *Clostridium difficile* Infection in Patients Hospitalized for Flare of Inflammatory Bowel Disease: A Retrospective Assessment. Dig. Liver Dis..

[23] Li Y., Xu H., Xu T., Xiao M., Tang H., Wu D., Tan B., Li J., Yang H., Lv H. (2018). Case–Control Study of Inflammatory Bowel Disease Patients with and without *Clostridium difficile* Infection and Poor Outcomes in Patients Coinfected with *C. difficile* and Cytomegalovirus. Dig. Dis. Sci..

[24] Song E.M., Choi A., Kim S., Jung S.H. (2023). The Prevalence and Risk Factors of *Clostridioides difficile* Infection in Inflammatory Bowel Disease: 10-Year South Korean Experience Based on the National Database. J. Korean Med. Sci..

[25] Amakye D., Ssentongo P., Patel S., Dalessio S., Kochhar S., Momin A., Clarke K. (2025). Global Patterns of *Clostridioides difficile* Infection in Patients with Inflammatory Bowel Disease: A Systematic Review and Meta-Analysis of Prevalence, Epidemiology, and Risk Factors. Crohn’s Colitis 360.

[26] Morgan X.C., Tickle T.L., Sokol H., Gevers D., Devaney K.L., Ward D.V., Reyes J.A., Shah S.A., LeLeiko N., Snapper S.B. (2012). Dysfunction of the Intestinal Microbiome in Inflammatory Bowel Disease and Treatment. Genome Biol..

[27] Rodríguez C., Romero E., Garrido-Sanchez L., Alcaín-Martínez G., Andrade R., Taminiau B., Daube G., García-Fuentes E. (2020). Microbiota Insights in *Clostridium difficile* Infection and Inflammatory Bowel Disease. Gut Microbes.

[28] Allegretti J.R., Kearney S., Li N., Bogart E., Bullock K., Gerber G.K., Bry L., Clish C.B., Alm E., Korzenik J.R. (2016). Recurrent *Clostridium difficile* Infection Associates with Distinct Bile Acid and Microbiome Profiles. Aliment. Pharmacol. Ther..

[29] Barbara G., Barbaro M.R., Fuschi D., Palombo M., Falangone F., Cremon C., Marasco G., Stanghellini V. (2021). Inflammatory and Microbiota-Related Regulation of the Intestinal Epithelial Barrier. Front. Nutr..

[30] Dong D., Su T., Chen W., Wang D., Xue Y., Lu Q., Jiang C., Ni Q., Mao E., Peng Y. (2023). *Clostridioides difficile* Aggravates Dextran Sulfate Solution (DSS)-Induced Colitis by Shaping the Gut Microbiota and Promoting Neutrophil Recruitment. Gut Microbes.

[31] Cook L., Wong M.Q., Rees W.D., Schick A., Lisko D.J., Lunken G.R., Wang X., Peters H., Oliveira L., Lau T. (2024). Dysregulated Immunity to *Clostridioides difficile* in IBD Patients Without a History of Recognized Infection. Inflamm. Bowel Dis..

[32] Kelly C.P., Kyne L. (2011). The Host Immune Response to *Clostridium difficile*. J. Med. Microbiol..

[33] Bibbò S., Lopetuso L.R., Ianiro G., Di Rienzo T., Gasbarrini A., Cammarota G. (2014). Role of Microbiota and Innate Immunity in Recurrent *Clostridium difficile* Infection. J. Immunol. Res..

[34] Bassotti G., Fruganti A., Stracci F., Marconi P., Fettucciari K. (2023). Cytotoxic Synergism of *Clostridioides difficile* Toxin B with Proinflammatory Cytokines in Subjects with Inflammatory Bowel Diseases. World J. Gastroenterol..

[35] Huang J., Kelly C.P., Bakirtzi K., Villafuerte Gálvez J.A., Lyras D., Mileto S.J., Larcombe S., Xu H., Yang X., Shields K.S. (2018). *Clostridium difficile* Toxins Induce VEGF-A and Vascular Permeability to Promote Disease Pathogenesis. Nat. Microbiol..

[36] Balram B., Battat R., Al-Khoury A., D’Aoust J., Afif W., Bitton A., Lakatos P.L., Bessissow T. (2019). Risk Factors Associated with *Clostridium difficile* Infection in Inflammatory Bowel Disease: A Systematic Review and Meta-Analysis. J. Crohn’s Colitis.

[37] Vitikainen K., Kase M., Meriranta L., Molander P., Af Björkesten C.-G., Anttila V.-J., Arkkila P. (2025). Higher Disease Activity of Inflammatory Bowel Disease Predisposes to *Clostridioides difficile* Infection. Ther. Adv. Gastroenterol..

[38] Chen X.L., Deng J., Chen X., Wan S.S., Wang Y., Cao Q. (2019). High Incidence and Morbidity of *Clostridium difficile* Infection among Hospitalized Patients with Inflammatory Bowel Disease: A Prospective Observational Cohort Study. J. Dig. Dis..

[39] Martínez-Lozano H., Saralegui-Gonzalez P., Reigadas E., Fueyo-Peláez P.R., García-García A., Miranda-Bautista J., Alcalá L., Nieto J.C., Lobato-Matilla M.E., Marín-Jiménez I. (2025). Risk Factors for *Clostridioides difficile* Infection among Patients Diagnosed with Inflammatory Intestinal and Rheumatological Diseases in the Biologic Era. BMC Gastroenterol..

[40] Jakubowska A., Szydlarska D., Rydzewska G. (2024). Analysis of Risk Factors of *Clostridioides difficile* Infection in Patients with Inflammatory Bowel Disease. Przegląd Gastroenterol..

[41] Zhang T., Lin Q.-Y., Fei J.-X., Zhang Y., Lin M.-Y., Jiang S.-H., Wang P., Chen Y. (2016). *Clostridium difficile* Infection Worsen Outcome of Hospitalized Patients with Inflammatory Bowel Disease. Sci. Rep..

[42] Schneeweiss S., Korzenik J., Solomon D.H., Canning C., Lee J., Bressler B. (2009). Infliximab and Other Immunomodulating Drugs in Patients with Inflammatory Bowel Disease and the Risk of Serious Bacterial Infections. Aliment. Pharmacol. Ther..

[43] Bossuyt P., Verhaegen J., Van Assche G., Rutgeerts P., Vermeire S. (2009). Increasing Incidence of *Clostridium difficile*-Associated Diarrhea in Inflammatory Bowel Disease. J. Crohn’s Colitis.

[44] Issa M., Vijayapal A., Graham M.B., Beaulieu D.B., Otterson M.F., Lundeen S., Skaros S., Weber L.R., Komorowski R.A., Knox J.F. (2007). Impact of *Clostridium difficile* on Inflammatory Bowel Disease. Clin. Gastroenterol. Hepatol..

[45] Colombel J.-F., Sands B.E., Rutgeerts P., Sandborn W., Danese S., D’Haens G., Panaccione R., Loftus E.V., Sankoh S., Fox I. (2017). The Safety of Vedolizumab for Ulcerative Colitis and Crohn’s Disease. Gut.

[46] Chen W., Liu Y., Zhang Y., Zhang H., Chen C., Zhu S., Zhou Y., Zhao H., Zong Y. (2024). Risk of *Clostridioides difficile* Infection in Inflammatory Bowel Disease Patients Undergoing Vedolizumab Treatment: A Systematic Review and Meta-Analysis. BMC Gastroenterol..

[47] Sandborn W.J., Feagan B.G., Danese S., O’Brien C.D., Ott E., Marano C., Baker T., Zhou Y., Volger S., Tikhonov I. (2021). Safety of Ustekinumab in Inflammatory Bowel Disease: Pooled Safety Analysis of Results from Phase 2/3 Studies. Inflamm. Bowel Dis..

[48] Loftus E.V., Baumgart D.C., Gecse K., Kinnucan J.A., Connelly S.B., Salese L., Su C., Kwok K.K., Woolcott J.C., Armuzzi A. (2023). *Clostridium difficile* Infection in Patients with Ulcerative Colitis Treated with Tofacitinib in the Ulcerative Colitis Program. Inflamm. Bowel Dis..

[49] Voth E., Solanky D., Loftus E.V., Pardi D.S., Khanna S. (2021). Novel Risk Factors and Outcomes in Inflammatory Bowel Disease Patients with *Clostridioides difficile* Infection. Ther. Adv. Gastroenterol..

[50] Lowe S.C., Sauk J.S., Limketkai B.N., Kwaan M.R. (2021). Declining Rates of Surgery for Inflammatory Bowel Disease in the Era of Biologic Therapy. J. Gastrointest. Surg..

[51] Bagdasarian N., Rao K., Malani P.N. (2015). Diagnosis and Treatment of *Clostridium difficile* in Adults: A Systematic Review. JAMA.

[52] Spinelli A., Bonovas S., Burisch J., Kucharzik T., Adamina M., Annese V., Bachmann O., Bettenworth D., Chaparro M., Czuber-Dochan W. (2022). ECCO Guidelines on Therapeutics in Ulcerative Colitis: Surgical Treatment. J. Crohn’s Colitis.

[53] Adamina M., Minozzi S., Warusavitarne J., Buskens C.J., Chaparro M., Verstockt B., Kopylov U., Yanai H., Vavricka S.R., Sigall-Boneh R. (2024). ECCO Guidelines on Therapeutics in Crohn’s Disease: Surgical Treatment. J. Crohn’s Colitis.

[54] Seril D.N., Shen B. (2014). *Clostridium difficile* Infection in the Postcolectomy Patient. Inflamm. Bowel Dis..

[55] Kistangari G., Lopez R., Shen B. (2017). Frequency and Risk Factors of *Clostridium difficile* Infection in Hospitalized Patients with Pouchitis: A Population-Based Study. Inflamm. Bowel Dis..

[56] Barnes E.L., Agrawal M., Syal G., Ananthakrishnan A.N., Cohen B.L., Haydek J.P., Al Kazzi E.S., Eisenstein S., Hashash J.G., Sultan S.S. (2024). AGA Clinical Practice Guideline on the Management of Pouchitis and Inflammatory Pouch Disorders. Gastroenterology.

[57] Li Y., Qian J., Queener E., Shen B. (2013). Risk Factors and Outcome of PCR-Detected *Clostridium difficile* Infection in Ileal Pouch Patients. Inflamm. Bowel Dis..

[58] Navaneethan U., Giannella R.A. (2009). Thinking beyond the Colon-Small Bowel Involvement in *Clostridium difficile* Infection. Gut Pathog..

[59] Crobach M.J.T., Baktash A., Duszenko N., Kuijper E.J., Mastrantonio P., Rupnik M. (2018). Diagnostic Guidance for *C. difficile* Infections. Updates on Clostridium difficile in Europe.

[60] Senok A., Aldosari K., Alowaisheq R., Abid O., Alsuhaibani K., Khan M., Somily A. (2017). Detection of *Clostridium difficile* Antigen and Toxin in Stool Specimens: Comparison of the *C. difficile* Quik Chek Complete Enzyme Immunoassay and GeneXpert *C. difficile* Polymerase Chain Reaction Assay. Saudi J. Gastroenterol..

[61] Gateau C., Couturier J., Coia J., Barbut F. (2018). How to: Diagnose Infection Caused by *Clostridium difficile*. Clin. Microbiol. Infect..

[62] Prosty C., Hanula R., Katergi K., Longtin Y., McDonald E.G., Lee T.C. (2024). Clinical Outcomes and Management of NAAT-Positive/Toxin-Negative *Clostridioides difficile* Infection: A Systematic Review and Meta-Analysis. Clin. Infect. Dis..

[63] Park J., Kim S., Im J.P., Lee H.J., Kim J.S., Park H., Han Y.M., Koh S.-J. (2025). Clinical Outcome of Inflammatory Bowel Disease with *Clostridioides difficile* Polymerase Chain Reaction Toxin-Positive/Enzyme Immunoassay Toxin-Negative: A Retrospective Cohort Study. Dig. Dis. Sci..

[64] Turner D.P., Thorburn S.J., Crowe A., Jardine D., Timmins C. (2021). Trends in *Clostridioides difficile* Diagnosis before and after a Change in Testing Algorithm. J. Microbiol. Methods.

[65] Dbeibo L., Lucky C.W., Fadel W.F., Sadowski J., Beeler C., Kelley K., Williams J., Webb D., Kara A. (2023). Two-Step Algorithm-Based *Clostridioides difficile* Testing as a Tool for Antibiotic Stewardship. Clin. Microbiol. Infect..

[66] Ananthakrishnan A.N., Guzman-Perez R., Gainer V., Cai T., Churchill S., Kohane I., Plenge R.M., Murphy S. (2012). Predictors of Severe Outcomes Associated with *Clostridium difficile* Infection in Patients with Inflammatory Bowel Disease. Aliment. Pharmacol. Ther..

[67] Di Bella S., Di Masi A., Turla S., Ascenzi P., Gouliouris T., Petrosillo N. (2015). The Protective Role of Albumin in *Clostridium difficile* Infection: A Step Toward Solving the Puzzle. Infect. Control Hosp. Epidemiol..

[68] Abt M.C., McKenney P.T., Pamer E.G. (2016). *Clostridium difficile* Colitis: Pathogenesis and Host Defence. Nat. Rev. Microbiol..

[69] Ramachandran I., Sinha R., Rodgers P. (2006). Pseudomembranous Colitis Revisited: Spectrum of Imaging Findings. Clin. Radiol..

[70] Calméjane L., Laharie D., Kirchgesner J., Uzzan M. (2023). Review Article: Updated Management of Acute Severe Ulcerative Colitis: From Steroids to Novel Medical Strategies. UEG J..

[71] Kirkpatrick I.D.C., Greenberg H.M. (2001). Evaluating the CT Diagnosis of *Clostridium difficile* Colitis: Should CT Guide Therapy?. Am. J. Roentgenol..

[72] Autenrieth D.M., Baumgart D.C. (2012). Toxic Megacolon. Inflamm. Bowel Dis..

[73] Khanna S., Shin A., Kelly C.P. (2017). Management of *Clostridium difficile* Infection in Inflammatory Bowel Disease: Expert Review from the Clinical Practice Updates Committee of the AGA Institute. Clin. Gastroenterol. Hepatol..

[74] Solanky D., Pardi D.S., Loftus E.V., Khanna S. (2019). Colon Surgery Risk with Corticosteroids Versus Immunomodulators or Biologics in Inflammatory Bowel Disease Patients with *Clostridium difficile* Infection. Inflamm. Bowel Dis..

[75] Ben-Horin S., Margalit M., Bossuyt P., Maul J., Shapira Y., Bojic D., Chermesh I., Al-Rifai A., Schoepfer A., Bosani M. (2009). Combination Immunomodulator and Antibiotic Treatment in Patients with Inflammatory Bowel Disease and *Clostridium difficile* Infection. Clin. Gastroenterol. Hepatol..

[76] Bar-Yoseph H., Daoud H., Ben Hur D., Chowers Y., Waterman M. (2020). Does Early Corticosteroid Therapy Affect Prognosis in IBD Patients Hospitalized with *Clostridioides difficile* Infection?. Int. J. Colorectal Dis..

[77] Lukin D.J., Lawlor G., Hudesman D.P., Durbin L., Axelrad J.E., Passi M., Cavaliere K., Coburn E., Loftus M., Jen H. (2019). Escalation of Immunosuppressive Therapy for Inflammatory Bowel Disease Is Not Associated with Adverse Outcomes After Infection with *Clostridium difficile*. Inflamm. Bowel Dis..

[78] Kucharzik T., Ellul P., Greuter T., Rahier J.F., Verstockt B., Abreu C., Albuquerque A., Allocca M., Esteve M., Farraye F.A. (2021). ECCO Guidelines on the Prevention, Diagnosis, and Management of Infections in Inflammatory Bowel Disease. J. Crohn’s Colitis.

[79] Moran G.W., Gordon M., Sinopolou V., Radford S.J., Darie A.-M., Vuyyuru S.K., Alrubaiy L., Arebi N., Blackwell J., Butler T.D. (2025). British Society of Gastroenterology Guidelines on Inflammatory Bowel Disease in Adults: 2025. Gut.

[80] Johnson S., Louie T.J., Gerding D.N., Cornely O.A., Chasan-Taber S., Fitts D., Gelone S.P., Broom C., Davidson D.M., for the Polymer Alternative for CDI Treatment (PACT) investigators (2014). Vancomycin, Metronidazole, or Tolevamer for *Clostridium difficile* Infection: Results from Two Multinational, Randomized, Controlled Trials. Clin. Infect. Dis..

[81] Allegretti J.R., Marcus J., Storm M., Sitko J., Kennedy K., Gerber G.K., Bry L. (2020). Clinical Predictors of Recurrence After Primary *Clostridioides difficile* Infection: A Prospective Cohort Study. Dig. Dis. Sci..

[82] Vega A.D., Heil E.L., Blackman A.L., Banoub M., Kristie Johnson J., Leekha S., Claeys K.C. (2020). Evaluation of Addition of Intravenous Metronidazole to Oral Vancomycin Therapy in Critically Ill Patients with Non-Fulminant Severe *Clostridioides difficile* Infection. Pharmacotherapy.

[83] Rokas K.E.E., Johnson J.W., Beardsley J.R., Ohl C.A., Luther V.P., Williamson J.C. (2015). The Addition of Intravenous Metronidazole to Oral Vancomycin Is Associated with Improved Mortality in Critically Ill Patients with *Clostridium difficile* Infection. Clin. Infect. Dis..

[84] Cornely O.A., Crook D.W., Esposito R., Poirier A., Somero M.S., Weiss K., Sears P., Gorbach S. (2012). Fidaxomicin versus Vancomycin for Infection with *Clostridium difficile* in Europe, Canada, and the USA: A Double-Blind, Non-Inferiority, Randomised Controlled Trial. Lancet Infect. Dis..

[85] Louie T.J., Miller M.A., Mullane K.M., Weiss K., Lentnek A., Golan Y., Gorbach S., Sears P., Shue Y.-K. (2011). Fidaxomicin versus Vancomycin for *Clostridium difficile* Infection. N. Engl. J. Med..

[86] Wolf J., Kalocsai K., Fortuny C., Lazar S., Bosis S., Korczowski B., Petit A., Bradford D., Croos-Dabrera R., Incera E. (2020). Safety and Efficacy of Fidaxomicin and Vancomycin in Children and Adolescents with *Clostridioides* (*Clostridium*) *difficile* Infection: A Phase 3, Multicenter, Randomized, Single-Blind Clinical Trial (SUNSHINE). Clin. Infect. Dis..

[87] Guery B., Menichetti F., Anttila V.-J., Adomakoh N., Aguado J.M., Bisnauthsing K., Georgopali A., Goldenberg S.D., Karas A., Kazeem G. (2018). Extended-Pulsed Fidaxomicin versus Vancomycin for *Clostridium difficile* Infection in Patients 60 Years and Older (EXTEND): A Randomised, Controlled, Open-Label, Phase 3b/4 Trial. Lancet Infect. Dis..

[88] Högenauer C., Mahida Y., Stallmach A., Marteau P., Rydzewska G., Ivashkin V., Gargalianos-Kakolyris P., Michon I., Adomakoh N., Georgopali A. (2018). Pharmacokinetics and Safety of Fidaxomicin in Patients with Inflammatory Bowel Disease and *Clostridium difficile* Infection: An Open-Label Phase IIIb/IV Study (PROFILE). J. Antimicrob. Chemother..

[89] Nerandzic M.M., Mullane K., Miller M.A., Babakhani F., Donskey C.J. (2012). Reduced Acquisition and Overgrowth of Vancomycin-Resistant Enterococci and Candida Species in Patients Treated with Fidaxomicin Versus Vancomycin for *Clostridium difficile* Infection. Clin. Infect. Dis..

[90] Rubio-Terrés C., Aguado J.M., Almirante B., Cobo J., Grau S., Salavert M., González Antona Sánchez E., López Gutiérrez C., Rubio-Rodríguez D. (2019). Extended-Pulsed Fidaxomicin versus Vancomycin in Patients 60 Years and Older with *Clostridium difficile* Infection: Cost-Effectiveness Analysis in Spain. Eur. J. Clin. Microbiol. Infect. Dis..

[91] Patel D., Senecal J., Spellberg B., Morris A.M., Saxinger L., Footer B.W., McDonald E.G., Lee T.C. (2022). Fidaxomicin to Prevent Recurrent *Clostridioides difficile*: What Will It Cost in the USA and Canada?. JAC-Antimicrob. Resist..

[92] Dilnessa T., Getaneh A., Hailu W., Moges F., Gelaw B. (2022). Prevalence and Antimicrobial Resistance Pattern of *Clostridium difficile* among Hospitalized Diarrheal Patients: A Systematic Review and Meta-Analysis. PLoS ONE.

[93] Toth M., Stewart N.K., Smith C., Vakulenko S.B. (2018). Intrinsic Class D β-Lactamases of *Clostridium difficile*. mBio.

[94] Sandhu B.K., Edwards A.N., Anderson S.E., Woods E.C., McBride S.M. (2019). Regulation and Anaerobic Function of the *Clostridioides difficile* β-Lactamase. Antimicrob. Agents Chemother..

[95] Darkoh C., Keita K., Odo C., Oyaro M., Brown E.L., Arias C.A., Hanson B.M., DuPont H.L. (2022). Emergence of Clinical *Clostridioides difficile* Isolates with Decreased Susceptibility to Vancomycin. Clin. Infect. Dis..

[96] Costa D.V.S., Pham N.V.S., Hays R.A., Bolick D.T., Goldbeck S.M., Poulter M.D., Hoang S.C., Shin J.H., Wu M., Warren C.A. (2022). Influence of Binary Toxin Gene Detection and Decreased Susceptibility to Antibiotics among *Clostridioides difficile* Strains on Disease Severity: A Single-Center Study. Antimicrob. Agents Chemother..

[97] Putsathit P., Hong S., George N., Hemphill C., Huntington P.G., Korman T.M., Kotsanas D., Lahra M., McDougall R., McGlinchey A. (2021). Antimicrobial Resistance Surveillance of *Clostridioides difficile* in Australia, 2015–2018. J. Antimicrob. Chemother..

[98] Saha S., Kapoor S., Tariq R., Schuetz A.N., Tosh P.K., Pardi D.S., Khanna S. (2019). Increasing Antibiotic Resistance in *Clostridioides difficile*: A Systematic Review and Meta-Analysis. Anaerobe.

[99] Olaitan A.O., Dureja C., Youngblom M.A., Topf M.A., Shen W.-J., Gonzales-Luna A.J., Deshpande A., Hevener K.E., Freeman J., Wilcox M.H. (2023). Decoding a Cryptic Mechanism of Metronidazole Resistance among Globally Disseminated Fluoroquinolone-Resistant *Clostridioides difficile*. Nat. Commun..

[100] Boekhoud I.M., Hornung B.V.H., Sevilla E., Harmanus C., Bos-Sanders I.M.J.G., Terveer E.M., Bolea R., Corver J., Kuijper E.J., Smits W.K. (2020). Plasmid-Mediated Metronidazole Resistance in *Clostridioides difficile*. Nat. Commun..

[101] Smits W.K., Harmanus C., Sanders I.M.J.G., Bry L., Blackwell G.A., Ducarmon Q.R., De Oliveira Ferreira E., Kuijper E.J. (2022). Sequence-Based Identification of Metronidazole-Resistant *Clostridioides difficile* Isolates. Emerg. Infect. Dis..

[102] Deshpande A., Wu X., Huo W., Palmer K.L., Hurdle J.G. (2020). Chromosomal Resistance to Metronidazole in *Clostridioides difficile* Can Be Mediated by Epistasis between Iron Homeostasis and Oxidoreductases. Antimicrob. Agents Chemother..

[103] Gargis A.S., Karlsson M., Paulick A.L., Anderson K.F., Adamczyk M., Vlachos N., Kent A.G., McAllister G., McKay S.L., Halpin A.L. (2023). Reference Susceptibility Testing and Genomic Surveillance of *Clostridioides difficile*, United States, 2012–2017. Clin. Infect. Dis..

[104] Shen W.-J., Deshpande A., Hevener K.E., Endres B.T., Garey K.W., Palmer K.L., Hurdle J.G. (2020). Constitutive Expression of the Cryptic vanGCd Operon Promotes Vancomycin Resistance in *Clostridioides difficile* Clinical Isolates. J. Antimicrob. Chemother..

[105] Belitsky B.R. (2022). VanG- and D-Ala-D-Ser-dependent Peptidoglycan Synthesis and Vancomycin Resistance in *Clostridioides difficile*. Mol. Microbiol..

[106] Tijerina-Rodríguez L., Villarreal-Treviño L., Baines S.D., Morfín-Otero R., Camacho-Ortíz A., Flores-Treviño S., Maldonado-Garza H., Rodríguez-Noriega E., Garza-González E. (2019). High Sporulation and Overexpression of Virulence Factors in Biofilms and Reduced Susceptibility to Vancomycin and Linezolid in Recurrent *Clostridium* [*Clostridioides*] *difficile* Infection Isolates. PLoS ONE.

[107] Buddle J.E., Thompson L.M., Williams A.S., Wright R.C.T., Durham W.M., Turner C.E., Chaudhuri R.R., Brockhurst M.A., Fagan R.P. (2024). Identification of Pathways to High-Level Vancomycin Resistance in *Clostridioides difficile* That Incur High Fitness Costs in Key Pathogenicity Traits. PLoS Biol..

[108] Thorpe C.M., McDermott L.A., Tran M.K., Chang J., Jenkins S.G., Goldstein E.J.C., Patel R., Forbes B.A., Johnson S., Gerding D.N. (2019). U.S.-Based National Surveillance for Fidaxomicin Susceptibility of *Clostridioides difficile*-Associated Diarrheal Isolates from 2013 to 2016. Antimicrob. Agents Chemother..

[109] Redmond S.N., Cadnum J.L., Jencson A.L., Kaple C.E., Wilson B.M., Skinner A.M., Gargis A.S., Hwang M., Choi H., Chatterjee P. (2025). Emergence and Spread of *Clostridioides difficile* Isolates with Reduced Fidaxomicin Susceptibility in an Acute Care Hospital. Clin. Infect. Dis..

[110] Marchandin H., Anjou C., Poulen G., Freeman J., Wilcox M., Jean-Pierre H., Barbut F. (2023). In Vivo Emergence of a Still Uncommon Resistance to Fidaxomicin in the Urgent Antimicrobial Resistance Threat *Clostridioides difficile*. J. Antimicrob. Chemother..

[111] Le T.M., Eubank T.A., McKelvey A.M., Cao X., Hurdle J.G., Garey K.W. (2024). Fidaxomicin Resistance in *Clostridioides difficile*: A Systematic Review and Predictive Modeling with RNA Polymerase Binding Sites. Antimicrob. Agents Chemother..

[112] Cao X., Boyaci H., Chen J., Bao Y., Landick R., Campbell E.A. (2022). Basis of Narrow-Spectrum Activity of Fidaxomicin on *Clostridioides difficile*. Nature.

[113] Schwanbeck J., Riedel T., Laukien F., Schober I., Oehmig I., Zimmermann O., Overmann J., Groß U., Zautner A.E., Bohne W. (2019). Characterization of a Clinical *Clostridioides difficile* Isolate with Markedly Reduced Fidaxomicin Susceptibility and a V1143D Mutation in rpoB. J. Antimicrob. Chemother..

[114] Baunwall S.M.D., Andreasen S.E., Hansen M.M., Kelsen J., Høyer K.L., Rågård N., Eriksen L.L., Støy S., Rubak T., Damsgaard E.M.S. (2022). Faecal Microbiota Transplantation for First or Second *Clostridioides difficile* Infection (EarlyFMT): A Randomised, Double-Blind, Placebo-Controlled Trial. Lancet Gastroenterol. Hepatol..

[115] Mullish B.H., Quraishi M.N., Segal J.P., McCune V.L., Baxter M., Marsden G.L., Moore D.J., Colville A., Bhala N., Iqbal T.H. (2018). The Use of Faecal Microbiota Transplant as Treatment for Recurrent or Refractory *Clostridium difficile* Infection and Other Potential Indications: Joint British Society of Gastroenterology (BSG) and Healthcare Infection Society (HIS) Guidelines. Gut.

[116] Ianiro G., Maida M., Burisch J., Simonelli C., Hold G., Ventimiglia M., Gasbarrini A., Cammarota G. (2018). Efficacy of Different Faecal Microbiota Transplantation Protocols for *Clostridium difficile* Infection: A Systematic Review and Meta-analysis. UEG J..

[117] Yadegar A., Bar-Yoseph H., Monaghan T.M., Pakpour S., Severino A., Kuijper E.J., Smits W.K., Terveer E.M., Neupane S., Nabavi-Rad A. (2024). Fecal Microbiota Transplantation: Current Challenges and Future Landscapes. Clin. Microbiol. Rev..

[118] Van Nood E., Vrieze A., Nieuwdorp M., Fuentes S., Zoetendal E.G., De Vos W.M., Visser C.E., Kuijper E.J., Bartelsman J.F.W.M., Tijssen J.G.P. (2013). Duodenal Infusion of Donor Feces for Recurrent *Clostridium difficile*. N. Engl. J. Med..

[119] Kelly C.R., Yen E.F., Grinspan A.M., Kahn S.A., Atreja A., Lewis J.D., Moore T.A., Rubin D.T., Kim A.M., Serra S. (2021). Fecal Microbiota Transplantation Is Highly Effective in Real-World Practice: Initial Results from the FMT National Registry. Gastroenterology.

[120] Baunwall S.M.D., Lee M.M., Eriksen M.K., Mullish B.H., Marchesi J.R., Dahlerup J.F., Hvas C.L. (2020). Faecal Microbiota Transplantation for Recurrent *Clostridioides difficile* Infection: An Updated Systematic Review and Meta-Analysis. eClinicalMedicine.

[121] Qazi T., Amaratunga T., Barnes E.L., Fischer M., Kassam Z., Allegretti J.R. (2017). The Risk of Inflammatory Bowel Disease Flares after Fecal Microbiota Transplantation: Systematic Review and Meta-Analysis. Gut Microbes.

[122] Tariq R., Disbrow M.B., Dibaise J.K., Orenstein R., Saha S., Solanky D., Loftus E.V., Pardi D.S., Khanna S. (2020). Efficacy of Fecal Microbiota Transplantation for Recurrent *C. difficile* Infection in Inflammatory Bowel Disease. Inflamm. Bowel Dis..

[123] Hirten R.P., Grinspan A., Fu S.-C., Luo Y., Suarez-Farinas M., Rowland J., Contijoch E.J., Mogno I., Yang N., Luong T. (2019). Microbial Engraftment and Efficacy of Fecal Microbiota Transplant for *Clostridium difficile* in Patients with and Without Inflammatory Bowel Disease. Inflamm. Bowel Dis..

[124] Newman K.M., Rank K.M., Vaughn B.P., Khoruts A. (2017). Treatment of Recurrent *Clostridium difficile* Infection Using Fecal Microbiota Transplantation in Patients with Inflammatory Bowel Disease. Gut Microbes.

[125] DeFilipp Z., Bloom P.P., Torres Soto M., Mansour M.K., Sater M.R.A., Huntley M.H., Turbett S., Chung R.T., Chen Y.-B., Hohmann E.L. (2019). Drug-Resistant E. Coli Bacteremia Transmitted by Fecal Microbiota Transplant. N. Engl. J. Med..

[126] Quera R., Espinoza R., Estay C., Rivera D. (2014). Bacteremia as an Adverse Event of Fecal Microbiota Transplantation in a Patient with Crohn’s Disease and Recurrent *Clostridium difficile* Infection. J. Crohn’s Colitis.

[127] Tixier E.N., Verheyen E., Luo Y., Grinspan L.T., Du C.H., Ungaro R.C., Walsh S., Grinspan A.M. (2022). Systematic Review with Meta-Analysis: Fecal Microbiota Transplantation for Severe or Fulminant *Clostridioides difficile*. Dig. Dis. Sci..

[128] Allegretti J.R., Feuerstadt P., Knapple W.L., Orenstein R., Pinton P., Sheh A., Khanna S. (2025). Safety and Efficacy of Fecal Microbiota, Live-Jslm (REBYOTA^®^), for the Prevention of Recurrent *Clostridioides difficile* Infection in Participants with Inflammatory Bowel Disease in PUNCH CD3-OLS. Inflamm. Bowel Dis..

[129] Ianiro G., Bibbò S., Porcari S., Settanni C.R., Giambò F., Curta A.R., Quaranta G., Scaldaferri F., Masucci L., Sanguinetti M. (2021). Fecal Microbiota Transplantation for Recurrent *C. difficile* Infection in Patients with Inflammatory Bowel Disease: Experience of a Large-Volume European FMT Center. Gut Microbes.

[130] Johnson S., Gerding D.N. (2019). Bezlotoxumab. Clin. Infect. Dis..

[131] Wilcox M.H., Gerding D.N., Poxton I.R., Kelly C., Nathan R., Birch T., Cornely O.A., Rahav G., Bouza E., Lee C. (2017). Bezlotoxumab for Prevention of Recurrent *Clostridium difficile* Infection. N. Engl. J. Med..

[132] Gerding D.N., Kelly C.P., Rahav G., Lee C., Dubberke E.R., Kumar P.N., Yacyshyn B., Kao D., Eves K., Ellison M.C. (2018). Bezlotoxumab for Prevention of Recurrent *Clostridium difficile* Infection in Patients at Increased Risk for Recurrence. Clin. Infect. Dis..

[133] Hernandez L.D., Kroh H.K., Hsieh E., Yang X., Beaumont M., Sheth P.R., DiNunzio E., Rutherford S.A., Ohi M.D., Ermakov G. (2017). Epitopes and Mechanism of Action of the *Clostridium difficile* Toxin A-Neutralizing Antibody Actoxumab. J. Mol. Biol..

[134] Posteraro B., Pea F., Masucci L., Posteraro P., Sanguinetti M. (2018). Actoxumab + Bezlotoxumab Combination: What Promise for *Clostridium difficile* Treatment?. Expert Opin. Biol. Ther..

[135] Tkaczyk C., Dayao D., Girouard D., Godfrey V., Gamson A., Stanley A.M., Stepanov O., DiGiandomenico A., Sellman B.R., Tzipori S. (2025). P-1055. Anti-Toxin B Neutralizing Monoclonal Antibody AZD5148 Provides Protection in a *Clostridioides difficile* Gnotobiotic Piglet Model. Open Forum Infect. Dis..

